# Senescent peritoneal mesothelium induces a pro-angiogenic phenotype in ovarian cancer cells in vitro and in a mouse xenograft model in vivo

**DOI:** 10.1007/s10585-015-9753-y

**Published:** 2015-10-03

**Authors:** Justyna Mikuła-Pietrasik, Patrycja Sosińska, Eryk Naumowicz, Konstantin Maksin, Hanna Piotrowska, Aldona Woźniak, Dariusz Szpurek, Krzysztof Książek

**Affiliations:** Department of Pathophysiology, Poznań University of Medical Sciences, Rokietnicka 8 Str., 60-806 Poznań, Poland; General Surgery Ward, Centrum Medyczne HCP, 28 czerwca 1956 r. 223/229 Str., 61-485 Poznań, Poland; Department of Clinical Pathology, Poznań University of Medical Sciences, Przybyszewskiego 49 Str., 60-355 Poznań, Poland; Department of Toxicology, Poznań University of Medical Sciences, Dojazd 30 Str., 60-631 Poznań, Poland; Division of Gynecological Surgery, Poznań University of Medical Sciences, Polna 33 Str., 60-535 Poznań, Poland

**Keywords:** Angiogenesis, Cellular senescence, Mesothelial cells, Ovarian cancer, Peritoneal cavity

## Abstract

It is believed that senescent cells contribute to the progression of primary and metastatic tumors, however, the exact mechanisms of this activity remain elusive. In this report we show that senescent human peritoneal mesothelial cells (HPMCs) alter the secretory profile of ovarian cancer cells (A2780, OVCAR-3, SKOV-3) by increasing the release of four angiogenic agents: CXCL1, CXCL8, HGF, and VEGF. Proliferation and migration of endothelial cells subjected to conditioned medium generated by: cancer cells modified by senescent HPMCs; cancer cells co-cultured with senescent HPMCs; and by early-passage HPMCs from aged donors, were markedly intensified. The same was the case for the vascularization, size and number of tumors that developed in the mouse peritoneum upon injection of ovarian cancer cells with senescent HPMCs. When the identified pro-angiogenic proteins were neutralized in conditioned medium from the cancer cells, both aspects of endothelial cell behavior intensified in vitro in response to senescent HPMCs were markedly reduced. The search for mediators of senescent HPMC activity using specific neutralizing antibodies and recombinant exogenous proteins showed that the intensified angiogenic potential of cancer cells was elicited by IL-6 and TGF-β1. At the transcriptional level, increased proliferation and migration of endothelial cells exposed to cancer cells modified by senescent HPMCs was regulated by HIF-1α, NF-κB/p50 and AP-1/c-Jun. Collectively, our findings indicate that senescent HPMCs may promote the progression of ovarian cancer cells by reprogramming their secretory phenotype towards increased production of pro-angiogenic agents and subsequent increase in the angiogenic capabilities of the vascular endothelium.

## Introduction

There is general agreement that senescent cells which accumulate in tissues during aging actively contribute to the development of a permissive tissue microenvironment in which pre-malignant and malignant cells acquire a more aggressive phenotype [1]. This property of senescent cells stems from their ability to secrete increased amounts of agents that disrupt normal tissue architecture, thus creating inflammation-like conditions and facilitating all critical aspects of cancer progression [2].

One of the most lethal age-associated malignancies is ovarian cancer [3]. In the late stages of the disease, cancer cells disseminate by ovarian surface shedding and colonize the peritoneal cavity. It has been found that the intraperitoneal spread of this malignancy is controlled by interactions between cancer cells and normal human peritoneal mesothelial cells (HPMCs) [4]. One of the most important elements of these interactions is the HPMCs’ ability to secrete various pro-angiogenic stimuli that contribute to the development of a niche in which cancer cells preferentially form tumors [5, 6]. Interestingly, the magnitude of pro-angiogenic agent release by the HPMCs has been found to be markedly increased when the cells become senescent [7]. Taking into account the fact that senescent HPMCs accumulate in the omentum during aging [8], these cells’ pro-angiogenic phenotype may have special importance in the increased aggressiveness of ovarian malignancy in elderly patients.

In this report we again focused on the pro-angiogenic capabilities of senescent HPMCs and we examined our original hypothesis which stated that these cells may support the expansion of ovarian cancer cells by altering their secretory profile towards increased production of autologous angiogenic agents. To this end, ovarian cancer cells were subjected to conditioned medium (CM) from young and senescent HPMCs, then the secretion of several soluble mediators of angiogenesis by these cells was analyzed. In order to check whether the altered cancer cell secretome is biologically relevant, CM obtained from: (i) cancer cells modified by young/senescent HPMCs; (ii) cancer cells co-cultured with young/senescent HPMCs; and (iii) cancer cells subjected to CM generated by young HPMCs derived from donors of varying ages, was applicated to primary endothelial cells to determine the efficacy of their proliferation and migration. In addition, the vascularization, size and number of tumors that developed in the mouse peritoneum upon injection of the cancer cells together with young or senescent HPMCs were examined. The phenomenological investigations were followed by mechanistic studies in which (a) senescent HPMC-derived mediator(s) of altered cancer cell secretory properties as well as signaling pathways activated in the cancer cells in response to these stimuli were identified.

## Materials and methods

### Chemicals

Unless otherwise stated, all chemicals were purchased from Sigma (St. Louis, MO). Cell culture plastics were from Nunc (Roskilde, Denmark). Recombinant human IL-6 and TGF-β1 were from R&D Systems (Abingdon, UK). Neutralizing antibodies as well as appropriate isotype-matched control antibodies were from R&D Systems and Abcam (Cambridge, UK). Bay 87-2243, MG-132 and 3-aminobenzamide (3-AB) were from Bayer Pharma AG (Wuppertal, Germany), Biomol (Plymouth Meeting, PA) and Sigma, respectively.

### Cell cultures

Human peritoneal mesothelial cells (HPMCs) were isolated from pieces of omentum, as described elsewhere [9]. The tissues were obtained from 22 patients undergoing elective abdominal surgery. The reasons for the surgery included: aortic aneurysm (12), hernia (5), bowel obstruction (3), and cholelithiasis (2). All cultures were established from individuals with no evidence of peritonitis, diabetes, uremia or peritoneal malignancy. The age of the donors ranged from 19 to 85 years old. Cells were identified as pure mesothelial by their typical cobblestone appearance at confluence and uniform positive staining for HBME-1 and Wt-1 antigens. The study was approved by an institutional ethics committee and was performed in accordance with the ethical standards as laid down in the 1964 Declaration of Helsinki. HPMCs were propagated in M199 medium with 10 % fetal bovine serum (FBS).

Senescence of HPMCs was induced by serial passaging until their ability to replicate was exhausted. Cells were considered senescent when they developed a hypertrophic morphology and failed to increase in number for 4 weeks [10]. Cells described as “young” were derived from the first passage. In all in vitro and in vivo experiments, young and senescent cells obtained exclusively from the same donors were compared.

Ovarian cancer cells A2780 and SKOV-3 were purchased from the ECCC (Porton Down, UK) and maintained in RPMI 1640 medium with l-glutamine (2 mmol/L), penicillin (100 U/ml), streptomycin (100 g/ml) and 10 % FBS. The OVCAR-3 line was obtained from the ATCC (Rockville, MD) and grown in RPMI 1640 with l-glutamine (2 mmol/L), HEPES (10 mmol/L), glucose (4500 mg/L), insulin (0.01 mg/ml), and 20 % FBS.

Human umbilical vein endothelial cells (HUVECs) were purchased in the ATCC. The cells were cultured in DMEM with 15 % FBS, l-glutamine (2 mM), HEPES (20 mM), EGF (10 µg/ml), heparin (5 U/ml), penicillin (100 U/ml), and streptomycin (100 µg/ml).

### Conditioned media

In order to generate conditioned medium (CM), young and senescent HPMCs were seeded into 25 cm^5^ flasks, allowed to attach for 4 h, and were incubated in serum-free medium for 72 h. The ovarian cancer cells were grown until reaching 80–90 % confluency and then were maintained in serum-free conditions for 24 h. Afterwards, the cells were incubated with CM produced by young/senescent HPMCs and with CM generated by intact cancer cells for the next 24 h. After incubation the media were removed, the cells were washed and then subjected to serum-free medium (for 24 h) to generate autologous CM. In some experiments the CM was also generated by cancer cells co-cultured with young or senescent HPMCs in an arbitrarily selected 1:1 ratio for 72 h. All conditioned media that were obtained were filtered and frozen at −80 °C until required.

### Examination of cell secretome

Concentrations of soluble agents in cell culture supernatants from young and senescent HPMCs and from ovarian cancer cells were measured with appropriate DuoSet^®^ Immunoassay Development kits (R&D Systems) according to the manufacturer’s instructions.

### Proliferation and migration assays

Endothelial cell proliferation was assessed in serum-free conditions on low-density cultures by using the radioisotope method [10]. Endothelial cell migration towards CM obtained from cancer cells was examined using Transwell inserts (Costar, Inc., NY, USA) [11]. In both assays, endothelial cell exposure to CM lasted 24 h.

In some experiments, endothelial cell activity was assessed upon their exposure to CM from cancer cells modified by CM from senescent HPMCs pre-incubated with antibodies against PAI-1 (20 µg/ml), u-PA (100 µg/ml), TGF-β1 (400 ng/ml), IL-6 (200 ng/ml), CXCL1 (10 µg/ml), CXCL8 (20 µg/ml), VEGF (5 µg/ml), sICAM-1 (25 µg/ml), and TSP-1 (5 µg/ml), or with appropriate control antibodies for 4 h with mixing. In other experiments, endothelial cell proliferation and migration were tested in the presence of CM from cancer cells pre-incubated with specific neutralizing antibodies against CXCL1 (10 µg/ml), CXCL8 (20 µg/ml), HGF (10 µg/ml), and VEGF (5 µg/ml), or with appropriate isotype-matched control antibodies for 4 h with mixing. Some experiments included the examination of endothelial cell behavior upon the cells’ exposure to CM from cancer cells pre-incubated with exogenous recombinant human IL-6 (5 ng/ml) and TGF-β1 (1 ng/ml).

### Animal studies

Experiments were performed on 6-week-old, immunocompromised Scid mice weighing 20–24 g (CB17/I cr-Prkdc/I crI coCrl; Charles River, Wilmington, MA, USA). The animals were housed (5/cage) in filter top cages equipped with HEPA filters (Tecniplast, Buguggiate, Italy). The room climate was maintained at a temperature of 22 ± 3 °C, 40–65 % relative humidity, with 10 air changes per hour. A commercial diet (ISO 9001 certified laboratory feed, total pathogen free, Altromin, Germany) and drinking water supplemented with amoxicillin at 2 g/1 liter (Taromentin, Polfa, Poland) were available ad libitum. After a passage of ovarian cancer cells and HPMCs, the animals were injected i.p. with the cancer cells (A2780, OVCAR-3, SKOV-3) alone or in combination with young or senescent HPMCs in 100 µl of sterile PBS. The ratio of implanted cancer cells to HPMCs was 1:1 (4 × 10^6^ cells per animal in total). The health status of the animals, including the progression of intraperitoneal tumors, was inspected every 2 days. When the disease produced visible symptoms of cachexia (after 21 days of the experiment), the animals were sacrificed by i.m. injection of ketamine (30–35 mg/kg) with xylazine (40–90 mg/kg). All procedures were performed in compliance with EU Directive 2010/63/EU and were approved by an institutional ethics committee.

After euthanasia, the peritoneal cavity of the experimental animals was inspected by two qualified pathomorphologists and one oncologist. All tumors that had developed in the peritoneum were excised, counted, measured and photographed. Afterwards, the tumors were fixed in 4 % paraformaldehyde, dehydrated in alcohol series, embedded in paraffin and cut into 4 μm sections in a microtome. In order to identify the cancerous tissue, standard H + E staining was performed. The tumor burden was quantified according to the lesions’ count (as the total tumor number) and to the calculation of total tumor volume (using the formula: 0.5 × width^2^ × length [12]).

### Tumor vascularization measurements

Tumor vascularization was examined according to immunohistochemical detection of endothelial cell markers, i.e. CD31 and CD34 [13]. In brief, the specimens were incubated with an antibody against CD31 (Leica Biosystems, Buffalo Grove, IL) and against CD34 (Santa Cruz Biotechnology, Santa Cruz, CA), both diluted in a ratio of 1:25. Planimetric analysis of the brown-stained area reflecting the presence of CD31/CD34-positive cells was conducted using ImageJ 1.47v (Wayne Rasband, National Institute of Health, USA). Six to eight × 100 fields covering almost the whole of each of the six sections per group were examined. The results were expressed as a percentage (%), and the whole area of a specimen was treated as 100 %.

### Transcription factor analysis

Activation of hypoxia-inducible factor-1 alpha (HIF-1α), nuclear factor kappaB p50 subunit (NF-κB/p50) and activating protein-1 c-Jun subunit (AP-1/c-Jun) was examined using TransAM^®^ kits (Active Motif, Carlsbad, CA), as per manufacturer’s instructions. In some experiments the cancer cells were pre-treated (for 2 h) with Bay 87-2243 (2 nM), MG-132 (10 µM) or 3-AB (1 mM), which are specific inhibitors of HIF-1α, NF-κB, and AP-1, respectively, and then the CM generated by these cells was applied to HUVECs in order to assess their proliferation and migration. In other experiments the activation of the transcription factors was tested in a dose–response (0–10 ng/ml) and time-course (0–72 h) regimen upon cancer cell exposure to exogenous recombinant forms of IL-6 and TGF-β1, or in the presence of CM from senescent HPMCs pre-incubated with neutralizing antibodies against IL-6 (200 ng/ml) and TGF-β1 (400 ng/ml) or appropriate isotype-matched controls.

### Statistics

Statistical analysis was performed using GraphPad Prism™ 5.00 software (GraphPad Software, San Diego, USA). The means were compared with the Wilcoxon and Mann–Whitney tests. Correlations were analyzed using the Spearman test. Results were expressed as means ± SD. Differences with a *P* value <0.05 were considered to be statistically significant.

## Results and discussion

It has recently been found that senescent cells, including fibroblasts and mesothelial cells (HPMCs), secrete increased amounts of agents to the environment that stimulate angiogenic activity of the vascular endothelium [7, 14]. Taking into account the fact that senescent cells have been recognized to promote the progression of multiple types of cancers both in vitro and in vivo [15–17], the pro-angiogenic capabilities of these cells seem to be of special clinical significance.

**Fig. 1:**
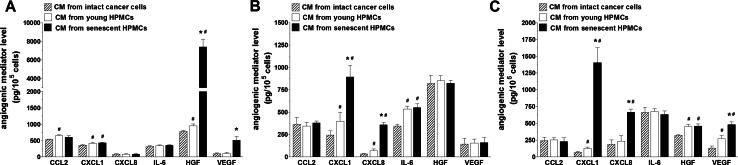
Effect of conditioned medium (CM) from young and senescent HPMCs on the secretion of angiogenesis mediators by A2780 (**a**), OVCAR-3 (**b**), and SKOV-3 (**c**) ovarian cancer cells. The *asterisks* indicate a significant difference as compared with cells exposed to the CM from young HPMCs. *Hashes* indicate a significant difference as compared with cells exposed to autologous CM from intact cancer cells. Experiments were performed with HPMCs from 8 different donors (samples were not pooled). The cancer cells were used in triplicates

In this report we verified our original hypothesis that senescent HPMCs may contribute to increased aggressiveness of ovarian cancer by increasing the pro-angiogenic capabilities of ovarian cancer cells. To this end, three representative lines of ovarian cancer cells, namely A2780, OVCAR-3, and SKOV-3 [18], were subjected to conditioned medium (CM) generated by young and senescent HPMCs, and then the secretion of six arbitrarily selected angiogenic agents, i.e. CCL2, CXCL1, CXCL8, HGF, IL-6, and VEGF [19, 20], by these cells was analyzed. The experiments depicted in Fig. 1 show that the senescent HPMCs were, indeed, capable of up-regulating the release of certain angiogenesis mediators by the cancer cells, albeit the pattern of this induction appeared to be specific for a given cell line; A2780 cells appeared to produce increased amounts of HGF and VEGF, OVCAR-3 cells increased the amounts of CXCL1 and CXCL8, while the SKOV-3 cells increased the amounts of CXCL1, CXCL8, and VEGF. It is likely that these diversified responses may reflect, at least partly, molecular differences among the investigated cell lines which, though of similar origin, appeared to differ at the genomic level [18].

**Fig. 2:**
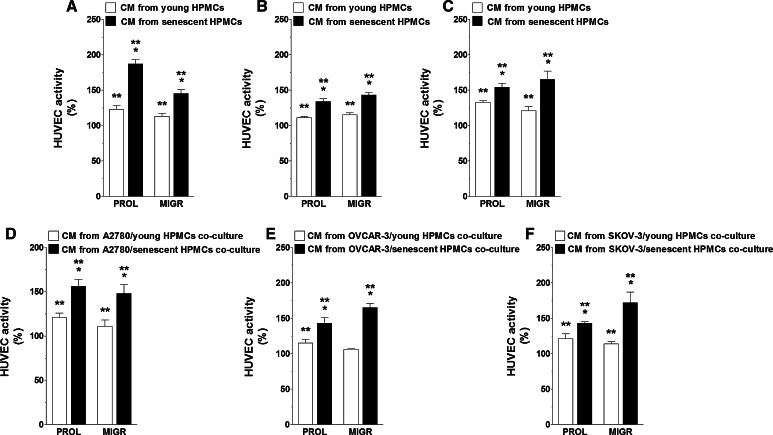
Modulatory effect of HPMCs on ovarian cancer cell-dependent angiogenic activity of endothelial cells in vitro. Both proliferation (PROL) and migration (MIGR) of endothelial cells (HUVECs) were examined in response to their exposure (24 h) to CM harvested from A2780 (**a**), OVCAR-3 (**b**), and SKOV-3 (**c**) ovarian cancer cells which were pre-incubated with CM from young and senescent HPMCs, or in response to exposure to CM generated by A2780 (**d**), OVCAR-3 (**e**), and SKOV-3 cells (**f**) co-cultured with young and senescent HPMCs. *Single* *asterisks* indicate a significant difference as compared with cells subjected to CM from cancer cells pre-incubated with CM from young HPMCs. *Double asterisks* indicate a significant difference as compared with cells subjected to CM from cancer cells pre-incubated with autologous CM (treated as 100 %). Experiments were performed with HPMCs from 8 different donors whose age ranged from 34 to 38 years old (samples were not pooled). Ovarian cancer cells were used in hexaplicates. Endothelial cells were used in duplicates

**Fig. 3:**
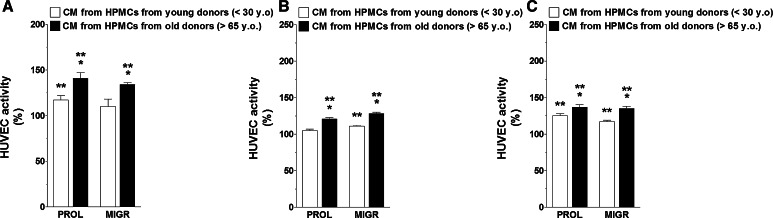
Modulatory effect of HPMCs on ovarian cancer cell-dependent angiogenic activity of endothelial cells in vitro. Both proliferation (PROL) and migration (MIGR) of endothelial cells (HUVECs) were examined in response to the cells’ exposure (24 h) to CM harvested from A2780 (**a**), OVCAR-3 (**b**), and SKOV-3 (**c**) ovarian cancer cells which were pre-incubated with CM from early-passage HPMCs isolated from young (<30 years old, n = 6) and old (>65 years old, n = 6) individuals. *Single asterisks* indicate a significant difference as compared with cells subjected to CM from cancer cells pre-incubated with CM from young donors. *Double asterisks* indicate a significant difference as compared with cells subjected to CM from cancer cells pre-incubated with autologous CM (treated as 100 %). Experiments were performed with HPMCs from 12 different donors whose age ranged from 19 to 85 years old (samples were not pooled). Ovarian cancer cells were used in hexaplicates. Endothelial cells were used in duplicates

Having established that senescent HPMCs increase the production of certain pro-angiogenic agents by ovarian cancer cells, primary cultures of endothelial cells (HUVECs) were subjected in parallel to CM obtained from cancer cells that had been pre-incubated with autologous CM and to CM generated by cancer cells modified by HPMCs. An analysis of angiogenic endothelial cell behavior showed that either the proliferation or migration of these cells was markedly improved when the cancer cells were subjected to the activity of senescent HPMCs (Fig. 2a–c). The same enhancement of endothelial cell motility was observed when HUVECs were exposed to CM produced by cancer cells co-cultured with senescent HPMCs (Fig. 2d–f). Interestingly, markedly increased proliferation and migration of the vascular endothelium was also recorded when the cells were exposed to CM from cancer cells undergoing pre-treatment with C generated by young cells established from aged (>65 years old) donors (vs. cells from patients <30 years old) (Fig. 3). The similarity of the results as depicted in Figs. 2 and 3 confirms our earlier suggestions that HPMCs isolated from aged individuals may consist of a considerable fraction of replicatively senescent cells which have the ability to impose some phenotypical features of senescence on the whole culture [21, 22]. Such a conclusion is in line with the observations of other authors who found that senescent fibroblasts modify the general characteristics of a culture when they constitute even only 10 % of the whole population [16].

Last but not least, the results obtained in these experiments confirmed that the mediators released by the cancer cells upon their modification by senescent HPMCs were biologically active. This conclusion stems from the observation that the angiogenic behavior of endothelial cells was significantly reduced when a CM generated by a given cancer cell line was pre-incubated with specific neutralizing antibodies directed against previously identified cell-specific pro-angiogenic stimuli (Table 1).

**Table 1:** Proliferation and migration of endothelial cells in response to CM from ovarian cancer cells modified by senescent HPMCs upon neutralization of individual angiogenesis mediators in cancer cell-derived CM

Neutralized mediator	HUVEC proliferation			HUVEC migration		
	A2780	OVCAR-3	SKOV-3	A2780	OVCAR-3	SKOV-3
CXCL1	98 ± 6	77 ± 5*	56 ± 1*	100 ± 3	95 ± 8	67 ± 7*
CXCL8	104 ± 8	88 ± 2*	89 ± 3*	104 ± 4	44 ± 2*	41 ± 3*
HGF	55 ± 6*	103 ± 7	98 ± 4	78 ± 3*	102 ± 3	99 ± 2
VEGF	65 ± 4*	102 ± 5	45 ± 2*	66 ± 1*	101 ± 4	45 ± 4*

The values are expressed as a percentage of endothelial cell proliferation/migration upon treatment with an intact CM from cancer cells pre-incubated with CM from senescent HPMCs (considered as 100 %). The concentrations of antibodies used here are provided in the "Materials and methods" section. The results derive from experiments performed with CM generated by HPMCs from 8 different donors. Cancer cells and endothelial cells were used in hexaplicates. The asterisks indicate a significant decrease in endothelial cell proliferation/migration

**Fig. 4:**
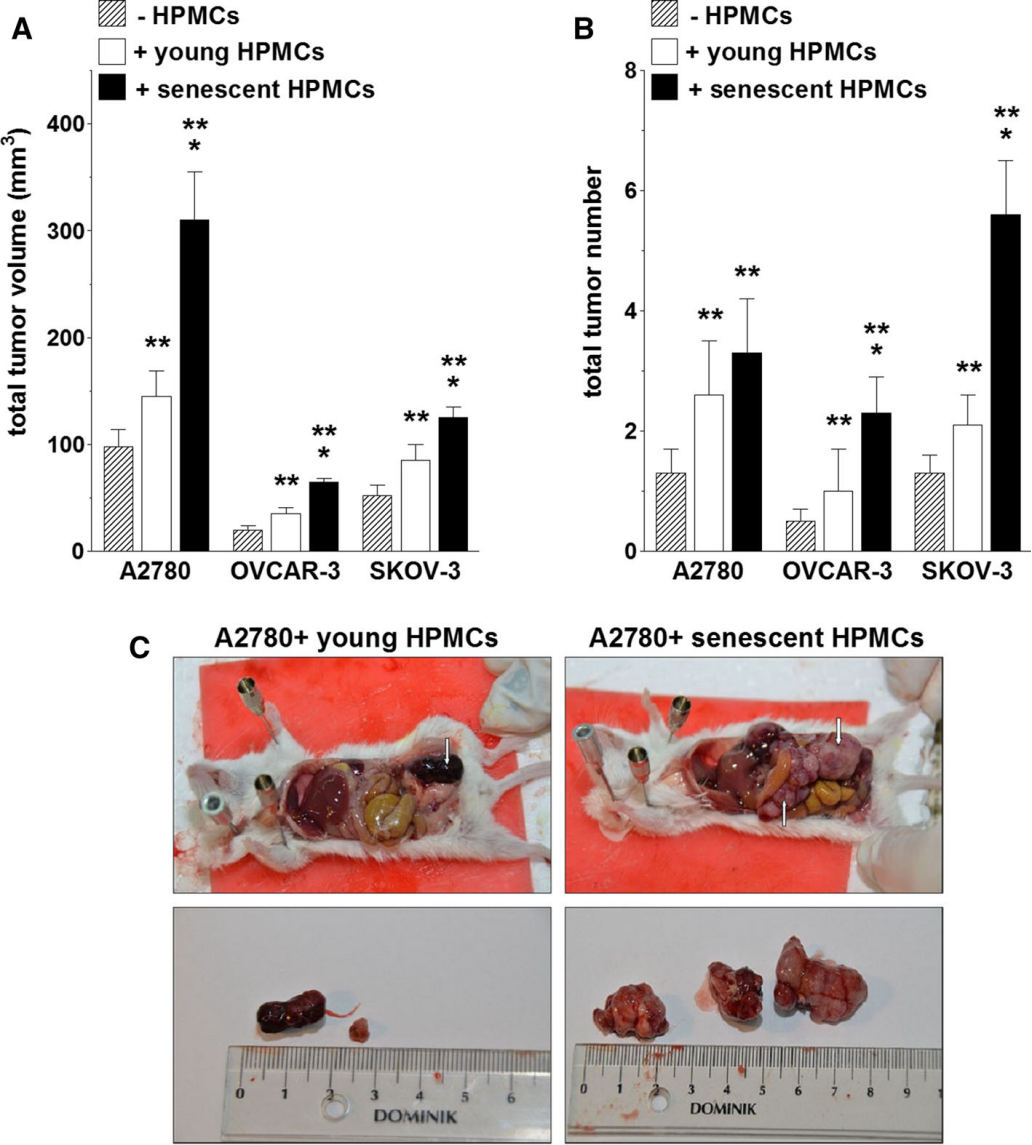
Development of tumors in the mouse peritoneal cavity upon injection of ovarian cancer cells with young and senescent HPMCs. Ovarian cancer cells (A2780, OVCAR-3, SKOV-3) alone or mixtures of ovarian cancer cells with respective HPMCs (1:1 ratio, 4 × 10^6^ cells in total) were injected intraperitoneally and the formation of xenografts was monitored for 21 days. The tumor burden was quantified according to the total tumor volume (**a**) and the total tumor number (**b**). Representative pictures showing tumors (marked with *arrows*) that developed upon the co-transplantation of A2780 cells together with young and senescent HPMCs (**c**). The *single asterisks* indicate a significant difference as compared with the xenografts that developed in the presence of young HPMCs. The *double asterisks* indicate a significant difference as compared with the xenografts that developed in the absence of HPMCs. Experiments were performed with HPMCs from 6 different donors and with 6 pairs of animals (each pair received cancer cells and young or senescent HPMCs from the same donor)

**Fig. 5:**
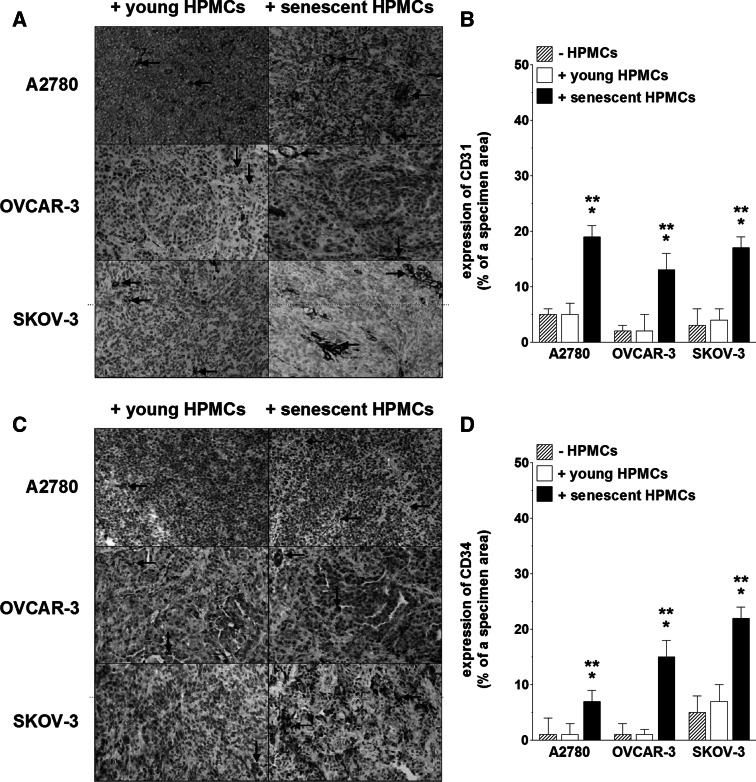
Magnitude of angiogenesis in tumors that developed upon i.p. injection of ovarian cancer cells alone or in combination with young and senescent HPMCs. The results derive from experiments as shown and described in Fig. 4. Representative images showing the results of immunostaining for markers of the vascular endothelium, CD31 (**a**) and CD34 (**c**). CD31/CD34-positive cells display brown membrane and/or cytoplasm staining (some examples are marked with *arrows*). Quantification of the brown-stained area reflecting the presence of CD31- (**b**) and CD34-positive cells (**d**). The results are expressed as a percentage, and the whole area of a specimen is considered as 100 %. The *single asterisks* indicate a significant difference as compared with the xenografts that developed in the presence of young HPMCs. The *double asterisks* indicate a significant difference as compared with the xenografts that developed in the absence of HPMCs. Experiments were performed with HPMCs from 6 different donors and with 6 pairs of animals (each pair received cancer cells and young or senescent HPMCs from the same donor)

The reinforced angiogenic potential of endothelial cells, initiated by senescent HPMCs and executed by the malignant cells, appeared to be valid also in in vivo conditions. The experiments showed that xenografts which had developed in the mouse peritoneum upon i.p. injection of ovarian cancer cells together with senescent HPMCs were significantly larger and their number was higher as compared with lesions fueled by young HPMCs (Fig. 4). It is likely that this effect was associated with an increased density of the microvessels, as evidenced according to immunohistochemistry against the endothelial cell markers CD31 and CD34 [13] (Fig. 5). These findings are in accordance with previous studies on mice which proved that effective angiogenesis is critical for successful development of ovarian tumors [23]. They are also in agreement with a report by Liu and Hornsby, who showed that breast cancer cells create solid tumors more effectively when they are cotransplanted in mice in combination with senescent fibroblasts [12]. Interestingly, also in this case the primary mechanism of the pro-cancerous activity of senescent cells was related to their impact on tumor vasculature, in particular a metalloproteinase-related increase in permeability, which led to increased inflow of cancer-promoting stimuli [12]. Last but not least, it is worth noting that increased tumor growth in animals co-injected with cancer cells with young HPMCs in comparison with those in which only ovarian cancer cells were implanted (Fig. 4) confirms our earlier hypothesis that the mesothelial cells themselves play an important cancer-promoting role in the early steps of intraperitoneal ovarian cancer metastasis [4]. At the same time, the lack of an analogical effect with respect to tumor vascularization (Fig. 5) may indicate that the stimulatory activity of young mesothelial cells towards ovarian cancer may be related to improved cancer cell adhesion and/or proliferation rather than to the strengthened angiogenic activity of endothelial cells.

Mechanistically, increased secretion of angiogenic factors by cancer cells subjected to senescent HPMCs has been found to be driven by two cytokines, IL-6 and TGF-β1, which have often been postulated as being involved in tumor neovascularization and in metastasis development efficiency [24]. Experiments have shown that among all of the soluble mediators released at an increased level by senescent HPMCs [25], only the neutralization of IL-6 and TGF-β1 in the CM from senescent cells led to a significant reduction of the pro-angiogenic activity of the cancer cells. When antibodies against the rest of the agents hypersecreted by senescent HPMCs, including CXCL1, CXCL8, ICAM-1, PAI-1, uPA, TSP-1, and VEGF, were used, both the proliferation and migration of the endothelial cells remained unchanged (Table 2). Moreover, the production of previously identified, cancer cell line-specific pro-angiogenic mediators (HGF, VEGF, CXCL1, and CXCL8) was markedly decreased when the CM generated by senescent HPMCs was pre-incubated with antibodies neutralizing IL-6 and TGF-β1 (Table 3).

**Table 2:** Proliferation and migration of endothelial cells in response to CM from ovarian cancer cells pre-incubated with CM from senescent HPMCs in which individual proteins were neutralized

Neutralized mediator	HUVEC proliferation			HUVEC migration		
	A2780	OVCAR-3	SKOV-3	A2780	OVCAR-3	SKOV-3
CXCL1	104 ± 5	111 ± 3	124 ± 12	105 ± 6	89 ± 11	89 ± 21
CXCL8	92 ± 6	92 ± 8	100 ± 4	103 ± 5	101 ± 8	102 ± 6
IL-6	72 ± 3*	64 ± 5*	68 ± 2*	64 ± 6*	81 ± 4*	78 ± 2*
PAI-1	113 ± 22	115 ± 21	104 ± 5	101 ± 1	105 ± 9	106 ± 6
u-PA	92 ± 11	95 ± 7	99 ± 1	98 ± 12	95 ± 5	101 ± 3
sICAM-1	107 ± 2	110 ± 8	96 ± 16	102 ± 11	103 ± 4	100 ± 8
TGF-β1	84 ± 5*	81 ± 2*	87 ± 4*	84 ± 2*	88 ± 1*	103 ± 8
TSP-1	106 ± 12	102 ± 5	102 ± 3	101 ± 1	112 ± 5	102 ± 7
VEGF	96 ± 3	91 ± 8	106 ± 8	98 ± 2	101 ± 1	92 ± 9

The values are expressed as a percentage of endothelial cell proliferation/migration upon incubation with CM from cancer cells pre-treated with an intact CM from senescent HPMCs (considered as 100 %). Please note that the neutralization of IL-6 and TGF-β1 decreased both the proliferation and migration of endothelial cells to a level characterizing their behavior upon exposure to CM generated by cancer cells pre-incubated with an autologous CM. The concentrations of all antibodies used here are provided in the "Materials and methods" section. The results derive from experiments performed with CM produced by HPMCs from 8 different donors. Cancer cells and endothelial cells were used in hexaplicates. The asterisks indicate a significant decrease in endothelial cell proliferation/migration

**Table 3:** Secretion of pro-angiogenic agents by ovarian cancer cells in response to a conditioned medium generated by senescent HPMCs upon its pre-incubation with antibodies neutralizing IL-6 and TGF-β1

Neutralized mediator	A2780		OVCAR-3		SKOV-3		
	HGF	VEGF	CXCL1	CXCL8	CXCL1	CXCL8	VEGF
IL-6	88 ± 3*	72 ± 4*	91 ± 2*	64 ± 3*	81 ± 2*	66 ± 3*	72 ± 1*
TGF-β1	76 ± 3*	82 ± 1*	78 ± 2*	76 ± 2*	80 ± 5*	71 ± 2*	67 ± 8*

Samples of CM from senescent HPMCs were pre-incubated with antibodies against IL-6 (200 ng/ml) and TGF-β1 (400 ng/ml) for 24 h. Afterwards, the media were added to ovarian cancer cells for 24 h. After incubation, the media were removed and the cells were exposed to fresh serum-free media for 24 h to generate autologous CM in which the angiogenic agent level was quantified. The values are expressed as a percentage of the control cells (cancer cells exposed to CM from senescent HPMCs without IL-6/TGF-β1 neutralization are considered as 100 %). The results derive from experiments performed with CM produced by HPMCs from 6 different donors. Cancer cells were used in hexaplicates. The asterisks indicate a significant difference as compared with the control group

**Fig. 6:**
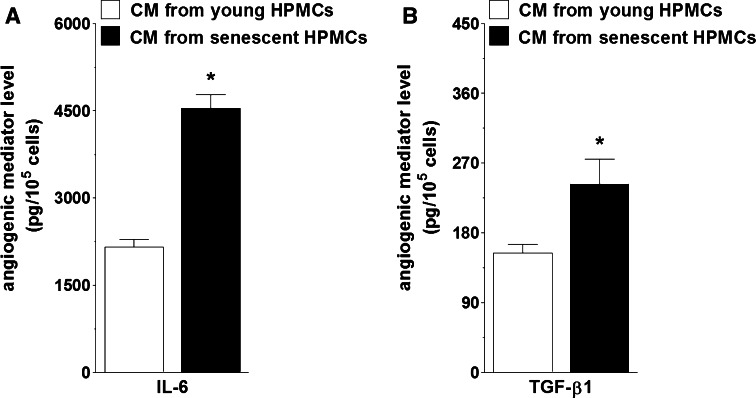
Changes in IL-6 (**a**) and TGF-β1 (**b**) production during senescence of primary HPMCs. The concentration of both cytokines was quantified in CM obtained from early-passage and senescent cells. *Asterisks* indicate a significant difference as compared with CM from young HPMCs. Experiments were performed with HPMCs from 22 different donors (samples were not pooled)

**Fig. 7:**
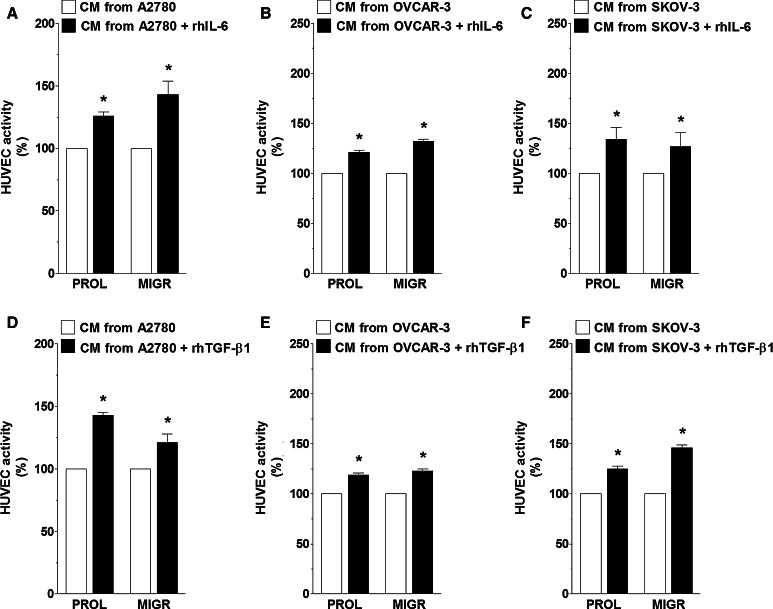
Effect of exogenous, recombinant forms of human IL-6 and TGF-β1 on ovarian cancer cell-dependent angiogenic reactions of endothelial cells. Both proliferation (PROL) and migration (MIGR) of endothelial cells (HUVECs) were examined in response to CM from A2780 (**a**, **d**), OVCAR-3 (**b**, **e**), and SKOV-3 cells (**c**, **f**) which were pre-incubated with recombinant human (rh) IL-6 (5 ng/ml) and TGF-β1 (1 ng/ml). *Asterisks* indicate a significant difference as compared with endothelial cells subjected to CM from cancer cells not exposed to rhIL-6 or rhTGF-β1 (treated as 100 %). Experiments were performed with CM obtained from 6 separate cultures of ovarian cancer cells (for each cell line). Endothelial cells were used in duplicates

In order to provide definitive evidence that the pro-angiogenic activity of mesothelium-derived IL-6 and TGF-β1 proceeds via stimulation of the release of specific angiogenic agents by ovarian cancer cells, we quantified both of these cytokines in the CM from young and senescent HPMCs and then correlated their level in the latter with the concentration of HGF, VEGF, CXCL1, and CXCL8 in the CM from A2780, OVCAR-3, and SKOV-3 cells. These analyses showed that, indeed, the senescence of primary HPMCs used in this study was associated with a significant increase in IL-6 and TGF-β1 secretion (Fig. 6), and that the higher their level in the CM from senescent cells was, the higher the production of angiogenesis mediators by cancer cells undergoing exposure to mesothelium-derived CM was (Table 4). The latter is in agreement with our further observations that both the proliferation and migration of endothelial cells were markedly intensified when the cells were exposed to CM from ovarian cancer cells, pre-incubated with exogenous recombinant forms of IL-6 and TGF-β1, and used at concentrations corresponding to their level in the CM from senescent HPMCs (Fig. 7).

**Table 4:** Correlation between the concentration of IL-6 and TGF-β1 in conditioned medium from senescent HPMCs and the concentration of HGF, VEGF, CXCL1, and CXCL8 in media generated by A2780, OVCAR-3, and SKOV-3 cells

Senescent HPMCs	A2780		OVCAR-3		SKOV-3		
	HGF	VEGF	CXCL1	CXCL8	CXCL1	CXCL8	VEGF
IL-6	P < 0.04 r = 0.541	P < 0.05 r = 0.621	P < 0.02 r = 0.744	P < 0.04 r = 0.641	P < 0.04 r = 0.523	P < 0.05 r = 0.671	P < 0.05 r = 0.473
TGF-β1	P < 0.02 r = 0.827	P < 0.05 r = 0.635	P < 0.05 r = 0.732	P < 0.03 r = 0.742	P < 0.02 r = 0.826	P < 0.05 r = 0.463	P < 0.04 r = 0.632

Ovarian cancer cells were exposed to CM obtained from senescent HPMCs for 24 h. Afterwards, the media were removed, the cells were washed and then subjected to serum-free medium (for 24 h) to generate autologous CM. Concentrations of IL-6 and TGF-β1 in the CM from senescent HPMCs were correlated with concentrations of HGF, VEGF, CXCL1, and CXCL8 quantified in cancer cell-derived CM using the Spearman test. The results derive from experiments performed with CM produced by HPMCs from 22 different donors

**Fig. 8:**
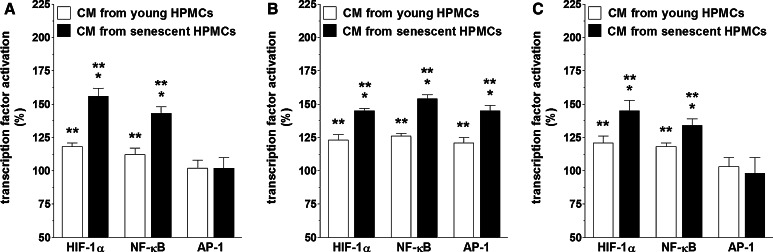
Activation of transcription factors involved in angiogenic endothelial cell reactions in ovarian cancer cells in response to senescence of HPMCs. Ovarian cancer cell lines A2780 (**a**), OVCAR-3 (**b**), and SKOV-3 (**c**) were subjected to conditioned medium from young and senescent HPMCs (24 h) and then the activation of HIF-1α, NF-κB, and AP-1 in the cancer cells was examined. *Single asterisks* indicate a significant difference as compared with cells exposed to CM from young HPMCs. *Double asterisks* indicate a significant difference as compared with cells subjected to autologous CM (treated as 100 %). Experiments were performed with HPMCs from 8 different donors (samples were not pooled). Ovarian cancer cells were used in hexaplicates

**Fig. 9:**
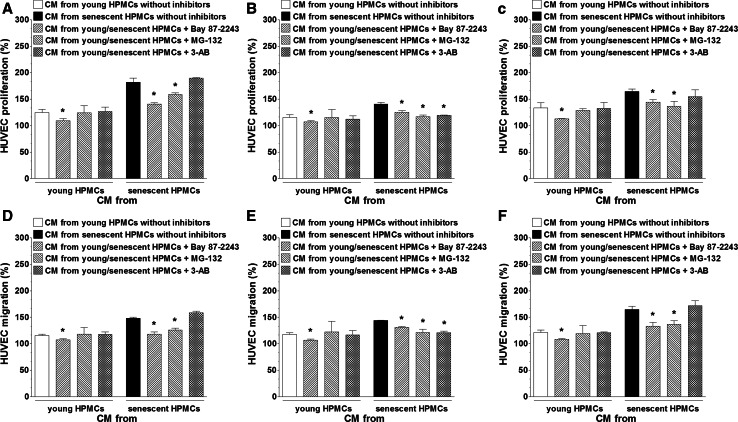
Transcriptional regulation of senescent HPMC-dependent stimulation of the pro-angiogenic activity of ovarian cancer cells. In the experiments, both the proliferation (*upper panel*) and migration (*bottom panel*) of endothelial cells (HUVECs) were evaluated upon their exposure (24 h) to CM from cancer cells (**a**, **d** A2780; **b**, **e** OVCAR-3; **c**, **f** SKOV-3), whose incubation with HPMC-derived CM was preceded by pre-treatment with Bay 87-2243, MG-132, and 3-AB which inhibit HIF-1α, NF-κB, and AP-1, respectively. The concentrations of inhibitors used here are provided in the see “Materials and methods” section. *Asterisks* indicate a significant difference as compared with cells exposed to CM from young or senescent HPMCs not treated with the transcription factor inhibitors. Experiments were performed with HPMCs from 8 different donors (samples were not pooled). Ovarian cancer cells were used in hexaplicates

**Fig. 10:**
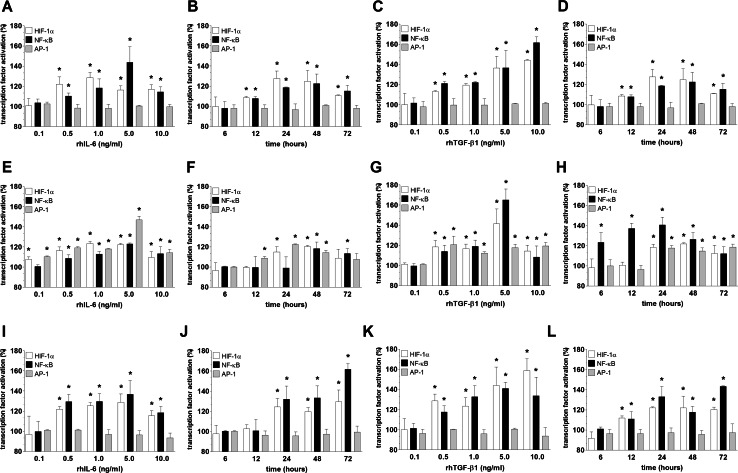
Effect of exogenous, human recombinant (rh) forms of IL-6 and TGF-β1 on the nuclear binding of HIF-1α, NF-κB, and AP-1 in ovarian cancer cells (**a**–**d** A2780; **e**–**h** OVCAR-3; **i**–**l** SKOV-3). Panels **a**, **c**, **e**, **g**, **i**, **k** show the results of dose–response experiments, while panels **b**, **d**, **f**, **h**, **j**, **l** represent the results of the time-course studies. Doses of recombinant proteins applied on the cancer cells (for 24 h) in the dose–response experiments correspond to a wide range of concentrations of a certain protein determined in conditioned medium generated by senescent HPMCs. In the time-course experiments, the lowest dose capable of inducing a given transcription factor was used. The *asterisks* indicate significant differences as compared with the control, untreated cells (100 %). The experiments were performed in hexaplicates

When it comes to the signaling pathways initiated in the cancer cells upon their exposure to HPMC-derived CM (read: IL-6 and TGF-β1) and underlying their sharpened pro-angiogenic potential, the activation of three transcription factors involved in angiogenesis, i.e. HIF-1α, NF-κB/p50, and AP-1/c-Jun [19, 26], was examined. The experiments showed that senescent HPMCs elicited the pro-angiogenic cancer cell response by increasing the transcriptional activity of HIF-1α, NF-κB/p50 (in A2780 and SKOV-3 cells), and AP-/c-Jun (in OVCAR-3 cells), which was revealed by an analysis of the nuclear binding efficacy of these factors (Fig. 8). This was confirmed when both the proliferation and migration of endothelial cells were studied upon cancer cell pre-incubation with transcription factor-specific inhibitors. Interestingly, when it comes to the effect of young HPMCs on cancer cell-dependent stimulation of angiogenesis, this activity was related exclusively to HIF-1α (Fig. 9). Further research showed that recombinant forms of both IL-6 and TGF-β1 are truly able to increase the nuclear binding of the studied transcription factors in both a dose- and time-dependent manner (Fig. 10), while neutralization of these proteins using specific antibodies in CM from senescent HPMCs significantly reduced their activation (not shown). These findings pointing to the interplay between IL-6 and TGF-β1 and pro-angiogenic transcription factor activity are in agreement with observations that have been made by other authors (e.g. [27, 28] ).

Taken together, our findings indicate that senescent HPMCs promote angiogenesis not only directly, i.e. by stimulating endothelial cell motility [7], but also indirectly, i.e. by modulating the secretory profile of the ovarian cancer cells. This phenomenon may constitute a new mechanism by which senescent HPMCs, known to accumulate in the peritoneum with age [8], may create a metastatic niche which allows ovarian cancer cells to acquire a more aggressive phenotype and thereby to more effectively colonize the peritoneal cavity.
